# Functional Marker Detection and Analysis on a Comprehensive Transcriptome of Large Yellow Croaker by Next Generation Sequencing

**DOI:** 10.1371/journal.pone.0124432

**Published:** 2015-04-24

**Authors:** Shijun Xiao, Zhaofang Han, Panpan Wang, Fang Han, Yang Liu, Jiongtang Li, Zhi Yong Wang

**Affiliations:** 1 Key Laboratory of Healthy Mariculture for the East China Sea, Ministry of Agriculture; Fisheries College, Jimei University, Xiamen, Fujian, China; 2 Chinese Academy of Fishery Sciences, Beijing, China; Temasek Life Sciences Laboratory, SINGAPORE

## Abstract

Large yellow croaker (*Larimichthys crocea*) is an important economic fish in China and Eastern Asia. Because of the exhaustive fishing and overdense aquaculture, the wild population and the mariculture of the species are facing serious challenges on germplasm degeneration and susceptibility to infectious disease agents. However, a comprehensive transcriptome from multi-tissues of the species has not been reported and functional molecular markers have not yet been detected and analyzed. In this work, we applied RNA-seq with the Illumina Hiseq2000 platform for a multi-tissue sample of large yellow croaker and assembled the transcriptome into 88,103 transcripts. Of them, 52,782 transcripts have been successfully annotated by nt/nr, InterPro, GO and KEGG database. Comparing with public fish proteins, we have found that 34,576 protein coding transcripts are shared in large yellow croaker with zebrafish, medaka, pufferfish, and stickleback. For functional markers, we have discovered 1,276 polymorphic SSRs and 261, 000 SNPs. The functional impact analysis of SNPs showed that the majority (~75%) of small variants cause synonymous mutations in proteins, followed by variations in 3' UTR region. The functional enrichment analysis illuminated that transcripts involved in DNA bindings, enzyme activities, and signal pathways prominently exhibit less single-nucleotide variants but genes for the constituent of the muscular tissue, the cytoskeleton, and the immunity system contain more frequent SNP mutations, which may reflect the structural and functional selections of the translated proteins. This is the first work for the high-throughput detection and analysis of functional polymorphic SSR and SNP markers in a comprehensive transcriptome of large yellow croaker. Our study provides valuable transcript sequence and functional marker resources for the quantitative trait locus (QTL) identification and molecular selection of the species in the research community.

## Introduction

Large yellow croaker (*Larimichthys crocea*) is one of the most important commercial marine fish species and is widely aqua-farmed in China [1]. The wild population of the species, mainly distributing in coastal regions of East Asia, underwent a severe collapse in 1970s due to overfishing and marine habitat destruction [2]. Recently, the aquaculture of large yellow croaker has become a crucial source for the fish market. However, because of the loss of genetic diversity, over-dense aquaculture and environment degradation, the wild population and aquaculture industry of large yellow croaker are facing tremendous challenges from germplasm degeneration and poor diseases resistance [3, 4]. Therefore, there is a great demand to breed new strains with superior economic traits, as well as better resistances against pathogens. In spit of that several progresses have been achieved for genetic improvements of the species, the underlying genetic bases of economic traits and mechanisms to infection resistance are still not thoroughly understood [5], largely due to a lack in both the amount and the systematical studies of genomic data for the species. Trascriptome sequences and functional markers are highly valuable resources for us to understand molecular genetic mechanisms and to perform molecular aided selection of large yellow croaker.

With the advent and rapid development of Next-Generation-Sequencing (NGS) technologies, RNA-seq has become a reliable and powerful tool to probe gene expressions in living cells [6]. Given its ability to sequence all transcript fragments in one experiment, scientists have recently extended the application of the high-throughput method to organisms in agriculture, including aquaculture animals [7, 8]. Nowadays, RNA-seq has been employed in a wide range of aquaculture studies, such as transcriptome survey [9, 10], molecular marker development [11–13], genetic map construction [14, 15] and gene differentially expression analysis [16]. As one of the most important economic marine fish in China, the transcriptome studies for large yellow croaker is still lacking. In 2010, Zhou *et.al* [17] performed Expressed Sequence Tags (ESTs) sequencing on gonad-specific and gonad-related genes for large yellow croaker and clustered 2,916 unique cDNAs from 3,535 ESTs. This would be an early large-scale investigation of transcripts in large yellow croaker. To obtain more genes, high-throughput RNA-seq was also carried out for the species in 2010 [5] and 2014 [18]. However, since those studies mainly focused on micro-organism infection of the species, the reported sequence data only from spleen tissue are hardly representative of a comprehensive transcriptome of large yellow croaker. Meanwhile, effective and stable markers, such as Simple Sequence Repeats (SSR) and Single-nucleotide polymorphism (SNP), play a crucial role in association studies and molecular aided selections [14, 19]. Although SSR have been developed from the limited number of expression sequence tags (ESTs) [20] and small fraction of genome sequences [21] in large yellow croaker, high-throughput polymorphic SSR and SNP marker development and analysis on large yellow croaker transcriptome are still missing. Given the now-widespread application of NGS, there is an outstanding need for systematic detection and investigation of the functional SSR and SNP markers within a comprehensive transcriptome of the species.

Here, we present a study of mRNA sequences from a tissue-mixed sample extracted from multi large yellow croaker at different developmental stages. After assembly and annotation, the transcriptome sequences were compared with public protein sequences of zebrafish, medaka, pufferfish and stickleback to identify shared proteins in large yellow croaker. Transcript expression level and open reading frame (ORF) have also been analyzed to provide systematic information for each transcript. In addition, to provide abundant functional marker resources for the research community, we have detected polymorphic SSRs and SNPs and annotated SNP mutations in protein coding transcripts. This work provides valuable transcriptome sequences and functional polymorphic SSR and SNP markers for the following experimental validation and artificial breeding programs of large yellow croaker.

## Methods

### Ethics Statement

This study was approved by the Animal Care and Use committee of Fisheries College of Jimei University.

### Tissue sampling and RNA isolation

Large yellow croaker samples were obtained from the breeding base of Jimei University in Ningde, Fujian, China. To cover expressed transcripts in various developmental stages, the sample was collected from embryos cells, larval, 11 juvenile and 2 adult (one male and one female) fish. Ocular, skin, muscle, gonadal, intestinal, liver, kidney, blood, gall and air bladder tissues from juvenile and adult large yellow croaker were mixed and stored in RNA later for following experiments. Total RNA was extracted from ∼50 mg composite sample with TRIZOL Reagent (Invitrogen, USA) and incubated for 1 h at 37°C with 10 units of DNaseI (Takara, China) to eliminate genomic DNA. The absorbance of 1.91 at 260 nm/280 nm and the RIN of 9.8 were obtained for the purified RNA sample by Nanodrop ND-1000 spectrophotometer (LabTech, USA) and 2100 Bioanalyzer (Agilent Technologies, USA), respectively.

### Library preparation and sequencing

The extracted mRNA were fragmented using divalent cations after the purification process. The first-strand cDNA was synthesized using random hexamer primers and SuperscriptTM III (Invitrogen TM, Carlsbad, CA, USA), followed by the second-strand cDNA synthesis using buffer, dNTPs, RnaseH and DNA polymerase I. Short fragments were then purified with a QiaQuick PCR extraction kit (Qiagen) and resolved with EB buffer for end reparation and poly(A) addition. After connecting to adapters, fragments with suitable size were used as templates for the following PCR amplification. The paired-end library was prepared following the manual of the Paired-End Sample Preparation Kit (Illumina). Finally, the library with a insert length of ∼150 bp was sequenced by Illumina HiSeqTM 2000 in 100PE mode (Illumina Inc., San Diego, CA, USA). The short reads were deposited in the NCBI Sequence Read Archive (SRA) under Accession number of SRR1509885.

### Transcriptome *de novo* assembly and annotation

The quality of the sequenced reads was assessed by FastQC v1.10.1 [22]. The transcriptome was *de novo* assembled from short reads using a de Bruijn graph method by Trinity r20130814 [23]. Before transcriptome assembly, all reads were normalized *in silico* by the Perl script normalize_by_kmer_coverage, with the maximum coverage for reads set to 50. The assembled transcripts were then subjected to cd-hit 4.5.4 [24] with a sequence identity threshold of 0.9 to eliminate sequence redundancy. Sequence length statistics of the assembled transcriptome were performed by our own python scripts. The final transcriptome sequences were searched against National Center for Biotechnology Information (NCBI) non-redundant nucleotide sequence (nt) and non-redundant protein (nr) database by local blastn and blastx programs [25] with an E-value threshold of 1 × 10^−5^. After that, the transcripts were then further annotated in InterPro, Gene Ontology (GO), EC (Enzyme Code), and Kyoto Encyclopedia of Genes and Genomes (KEGG) database with Blast2GO [26].

### Transcript expression profile analysis and ORF identification

RSEM 1.2.9 [27] was employed to estimate the expression level of each transcript. The alignment of the sequenced reads to the assembled transcriptome was performed by bowtie 1.0.1 [28], followed by the core process of RSEM [27], estimating the count of RNA-seq reads to each transcript [29]. We used FPKM (Fragments Per Kilobase of transcript per Million fragments mapped) [6] to measure the normalized expression value. The length of transcripts with FPKM larger than 1 were analyzed and compared with those of the total transcripts.

TransDecoder version rel16JAN2014 (http://transdecoder.sourceforge.net/) was used to identify transcripts with potential protein coding regions. We extracted all transcripts that satisfied the following criteria: 1) having an open reading frame (ORF) larger than 300 bp, and 2) the putative peptide having at least one hit to Pfam domain database [30]. The length of transcripts with predicted ORF were analyzed and compared with those of the total transcripts. Transcripts with potential ORF were uploaded into WebMGA [31] (http://weizhong-lab.ucsd.edu/metagenomic-analysis) and protein classifications were searched against Eukaryotic Orthologous Groups (KOG) database.

### Functional markers development and analysis

To develop polymorphic SSR markers, we performed variable number tandem repeat analysis on the transcriptome. First, all tandem repeats were scanned using Tandem Repeats Finder (TRF) [32]. A candidate repeat unit was scored as match (+2), mismatch (-7) and InDel (-7) for the base alignments with an average matching probability of 80%, InDel probability of 10%, alignment score ≥ 50. To eliminate non-genomic polyA tails, a number of stretches of (A/T)_*n*_ at transcript end were excluded in this work. Repeat numbers for each tandem repeat were rounded to the nearest whole number for distribution analysis. LobSTR was used for polymorphic SSR detection because of its good performance in previous reports [33]. In this work, only polymorphic SSR with a quality higher than 30 and covered by more than 10 reads were obtained to ensure the quality of the polymorphic SSR calling.

The assembled transcripts were scanned for SNP by BWA 0.7.6a [34], samtools 0.1.19 [35] and GATK 2.8-1 [36]. The SNP calling was proceeded by a short reads alignment carried out with BWA-MEM algorithm, followed by coordinate sort and duplicate marking by SortSam and MarkDuplicates programs in Picard tools 1.107 (picard.sourceforge.net), respectively. To improve the quality of SNP calling, we applied four steps to reduce the false SNP: 1) a local realignment process was performed after short read mapping; 2) base Quality Score Recalibration (BQSR) [36] was employed to adjust the accuracy of the Phred quality scores; 3) alignments with paired information and with a mapping quality higher than 30 were used for SNP calling with the UnifiedGenotyper function in GATK; 4) SNPs and InDels with a depth higher than 8 and a quality score higher than 60 were obtained for the next functional analysis.

### Functional enrichment for high SNP-rate transcripts

GO functional enrichment analysis of the top highest SNP-rate (SNP number/transcript length) transcripts were performed by two-tailed Fish’s exact test [37] with Benjamini & Hochberg false discovery rate (FDR) [38] in Blast2GO [26]. The top 800 SNP-rate transcripts were compared with the whole transcriptome to highlights over- and under-represented GO-terms. Statistical significance was qualified by the FDR of 0.05 in the study.

## Results and Discussions

### Transcriptome assembly and assessment

With the Illumina HiSeq2000 platform, we generated 32 million paired reads with a total of over 6.4 billion bases information. The cleaned paired-end reads, after trimming adapter and barcode, were used for the following analysis. Phred quality scores for each base in cleaned reads were assessed by FastQC [22] and more than 95%(90%) of the overall bases were found to have Phred quality larger than 20(30). The high sequencing quality ensured reliable transcript fragments for the transcriptome assembly. To reduce memory and processor requirements during transcriptome assembly, sequence normalization was performed to reduce redundancy in the cleaned reads. The cleaned and normalized reads were then subjected to *de novo* transcriptome assembly using Trinity package [23], resulting into 107,406 transcripts. An additional step with cd-hit was followed to remove redundancy in the assembled contigs, reducing the transcriptome to 88,103 transcripts.

The lengths of the assembled sequences in the transcriptome range from 201 to 28,304 bp, with a total and a N50 length as 86.9 Mb and 1,934 bp (Table 1), respectively. All of top 10 longest transcripts (range from 15.2 kb to 28.3 kb) were aligned to known genes from close fish species in the NCBI nt/nr database, such as titin, plectin and MYC binding proteins, which are traditionally recognized as huge proteins in organisms. The validation of those long transcripts demonstrates that the huge contigs in the transcriptome are not from the chimerical assembly of repeated fragments in genes.

**Table 1:** Transcript number and length statistics of the transcriptome assembly, transcripts with FPKM ≥ 1 and transcripts with ORF.

	total transcriptome	FPMK ≥ 1	with ORF
transcript number	88,103	41,986	34,025
shortest length (bp)	201	201	297
longest length (bp)	28,304	28,304	28,304
N50 (bp)	1,934	2,329	2,633

### Functional annotation

**Fig 1:**
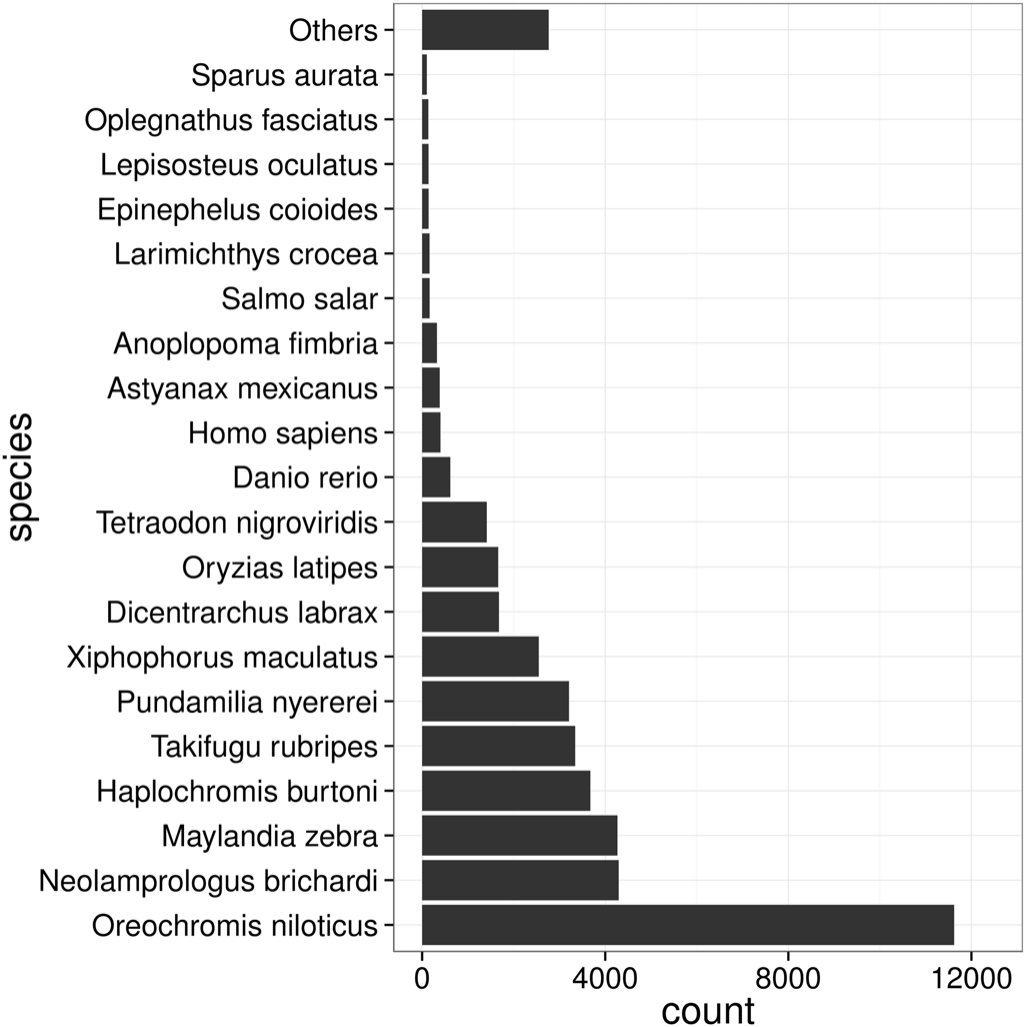
Species distribution for nr database blast. Note that only the best hits for transcripts are covered in the analysis.

To functionally annotate the transcriptome, we aligned the transcripts against NCBI nt/nr database to search for homologous sequences with BLAST applications. 45,733 (51.9%) and 42,360 (48.1%) transcripts showed significant similarity (E value ≤ 1 × 10^−5^) to nucleotide and protein sequences in NCBI nt/nr database, respectively. As shown in Fig 1, the top hits for a large fraction of transcripts (∼26.9%) were from Nile tilapia (*Oreochromis niloticus*), which can partially be explained by abundant sequence resources of that species and its close evolutionary relationship to large yellow croaker.

**Fig 2:**
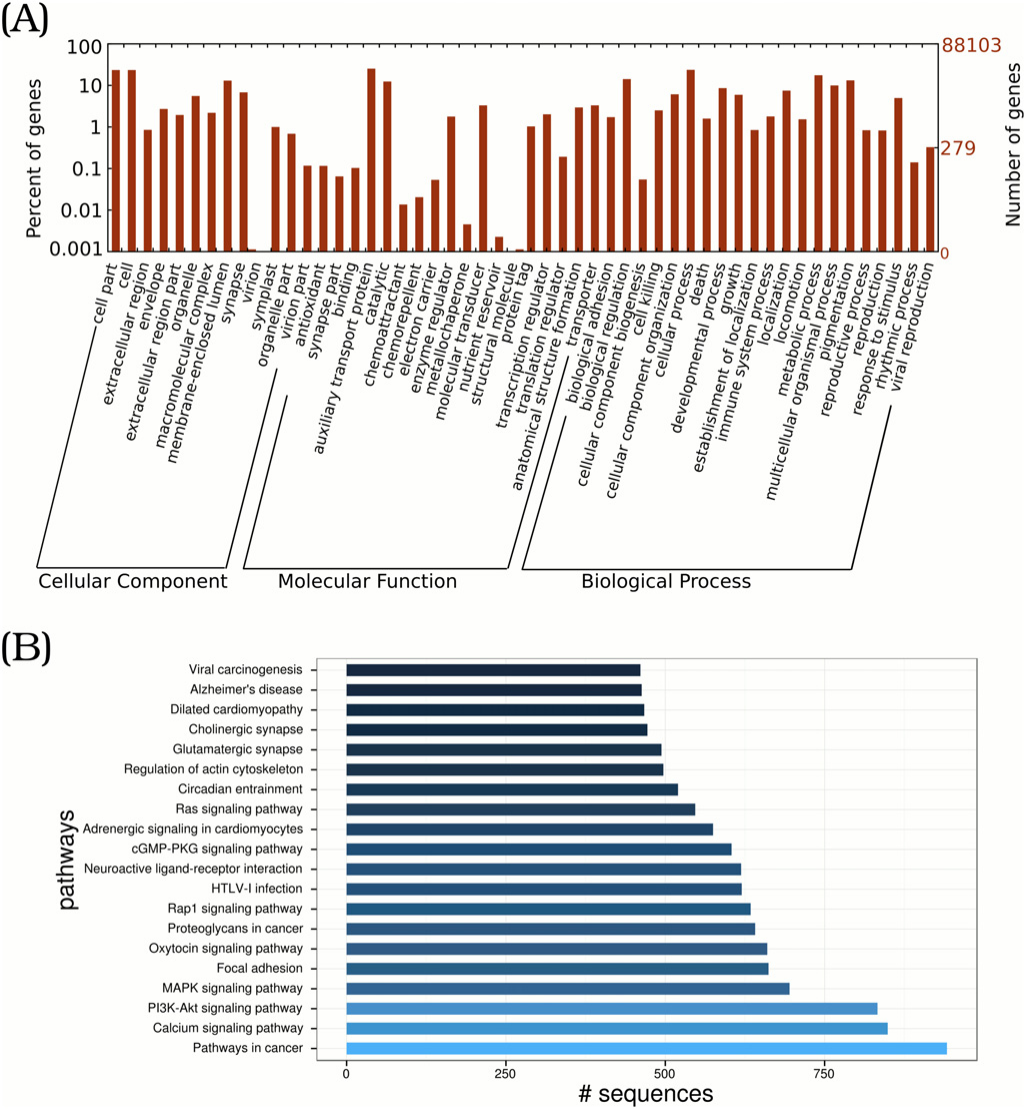
GO and KEGG classification of large yellow croaker transcriptome. Transcripts are annotated by Gene Ontology (GO) terms which belong to three main categories: biological process, cellular component, or molecular function. Only the top 20 most abundant pathways and the number of involved transcripts are listed.

In order to further annotate transcripts from gene products, the InterPro, Gene Ontology (GO) and Kyoto Encyclopedia of Genes and Genomes (KEGG) databases were used to identify functions of transcripts. We searched the transcriptome in 16 InterPro member databases, including HMMTigr, HMMPfam, FprinfScan, SuperFamily and SignalPHMM. 25,566 sequences have been annotated with InterPro accession numbers, accounting for ∼29% of total transcripts (Table 2). With parameters introduced in the Method section, we annotated transcripts to three major GO categories: Cell Component (CC), Molecular Function (MF), and Biological Process (BP). As listed in Table 2, 32,849 (37.3%) transcripts were associated with at least one GO term. For the second annotation level, 21,912 (24.9%), 28,761 (32.6%) and 25,823 (29.3%) sequences have been assigned to CC, MF and BP category, respectively. A summary of detailed annotations for each category is depicted in Fig 2(A) by WEGO application [39]. The most enriched components out of the CC terms are cell (GO:0005623) and cell part (GO:0044464). As for MF terms, a great number of transcripts are assigned to binding (GO:0005488), followed by catalytic activity (GO:0003824). In the BP category, cellular process (GO:0009987) and metabolic process (GO:0008152) are top two terms. According to GO-mapping results, ECs are assigned to 6,469 transcripts. By mapping transcripts to the reference canonical pathways in the KEGG database, 23,797 (27%) transcripts are assigned to KEGG Orthology (KO) terms and grouped into 328 pathways. The top enriched 20 pathways are shown in Fig 2(B). At present, we have identified 6,986 ECs from KEGG mapping results. From GO and KEGG mappings, ECs are assigned to 9,064 transcripts in total.

**Table 2:** Transcripts annotation by various databases. Note that the annotation for KOG are only performed for transcripts with predicted ORF.

database	hit number
nt	46,241
nr	43,044
InterPro	45,120
GO	32,849
KEGG	23,797
EC	9,064
KOG	23,753

### Compare with other fish protein database

To assess the assembled genes in our transcriptome with respect to close species, we aligned the transcriptome to the public zebrafish (*Danio rerio*) protein database v9.75 by blastx with the E-value threshold of 1 × 10^−3^. 38,943 (44.2%) transcripts searched hits. Detailed analysis found that many transcripts shared the same homologous targets, implying alternative splicing variants or assembly fragments from one gene. Omitting transcripts to the same target protein, we identified 34,330 protein-coding genes in large yellow croaker; the most abundant zebrafish protein found in large yellow croaker was titin protein domains (ENSDARP00000099532). By the same method, all sequences were also compared with the medaka (*Oryzias latipes*) 1.75, pufferfish (*Takifugu rubripes*) 4.75 and stickleback (*Gasterosteus aculeatus*) 1.75, respectively. 34,576 transcripts fall into the overlap of four comparisons, showing those genes that are shared those species (S1 Fig). Note that there are still many transcripts do not hit any target protein in the database, implying either non-coding RNA or species-specific genes. Therefore, the evaluation of transcript expression and ORF in sequences is crucial to further identify transcript functions.

### Transcripts expression analysis

In transcriptome, gene expression level is considered to be a valuable metrics for assessing the quality of transcript assembly, because transcripts with low expression level are more likely to be from immature primary transcripts or artificial transfrags [40]. Therefore, we estimated overall transcript expression level for each transcript to facilitate functional gene identification in large yellow croaker. To this end, the sequenced reads were mapped back to the transcriptome by Bowtie [28], and the count based method with RSEM [27] was applied to evaluate transcript expression. we found that FPMK of transcripts concentrated around 0.5∼2, as shown in S2 Fig. Expressions (FPKM) of top 50 most abundant transcripts were all higher than 2,500, and up to 25,000 for the highest one. Detailed functional analysis found that most of genes (> 40%) were 40S/60S ribosomal proteins, which was consistent with previous studies [41, 42]. Besides, many prevalent and crucial proteins were also discovered in the analysis, for examples apolipoprotein, chemotaxin, cytochrome c oxidase, pepsinogen, trypsinogen, lectin, fibrinogen.

### Protein coding region identification

**Fig 3:**
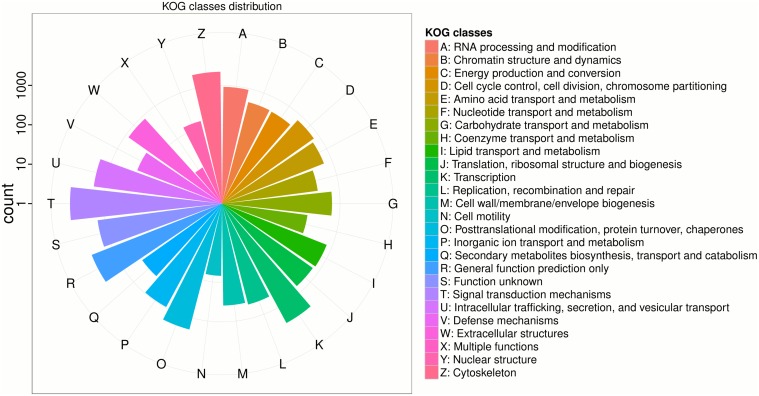
KOG categories for transcripts with ORF. Letters around the circle represents different KOG categories, as shown detail on right legend. The logarithm scaled radius for each group illuminates the transcript number involved in each category.

Researchers need a completed transcriptome to facilitate further qualitative and quantitative genetic studies. One method to assess the completeness of transcripts is to identify likely protein coding regions based on the already-existing protein sequence knowledge [43]. In this work, ORF sequences of each transcript were identified by searching Pfam database for homologous protein sequences with the TransDecoder package [30]. For single sequence with several possible ORFs, we only retained the longest one. By this method, we identified 34,025 reliable transcripts with significant ORF, with an N50 length of 2,633 bp (Table 1). Notably, we found the mean expression level for those transcripts to be as high as 20, which was significantly higher than the mean expression level (9) for the whole transcriptome. Those transcripts with potential ORFs were further annotated in WebMGA [31] (http://weizhong-lab.ucsd.edu/metagenomic-analysis) to predict Eukaryotic Orthologous Groups (KOG). More than 23,753 sequences were assigned to 26 KOG categories as depicted in Fig 3. As in the largest cluster, signal transduction mechanisms include 7518 transcripts, which is followed by general function prediction only (3,786) and transcription (2,874). We note that some of the identified bio-processes are directly related with the nutrition, meat quality and disease resistance of large yellow croaker, such as coenzyme transport and metabolism (169), lipid transport and metabolism(759) and amino acid transport and metabolism (622), secondary metabolites biosynthesis, transport and catabolism (314) and defense mechanisms (216).

**Fig 4:**
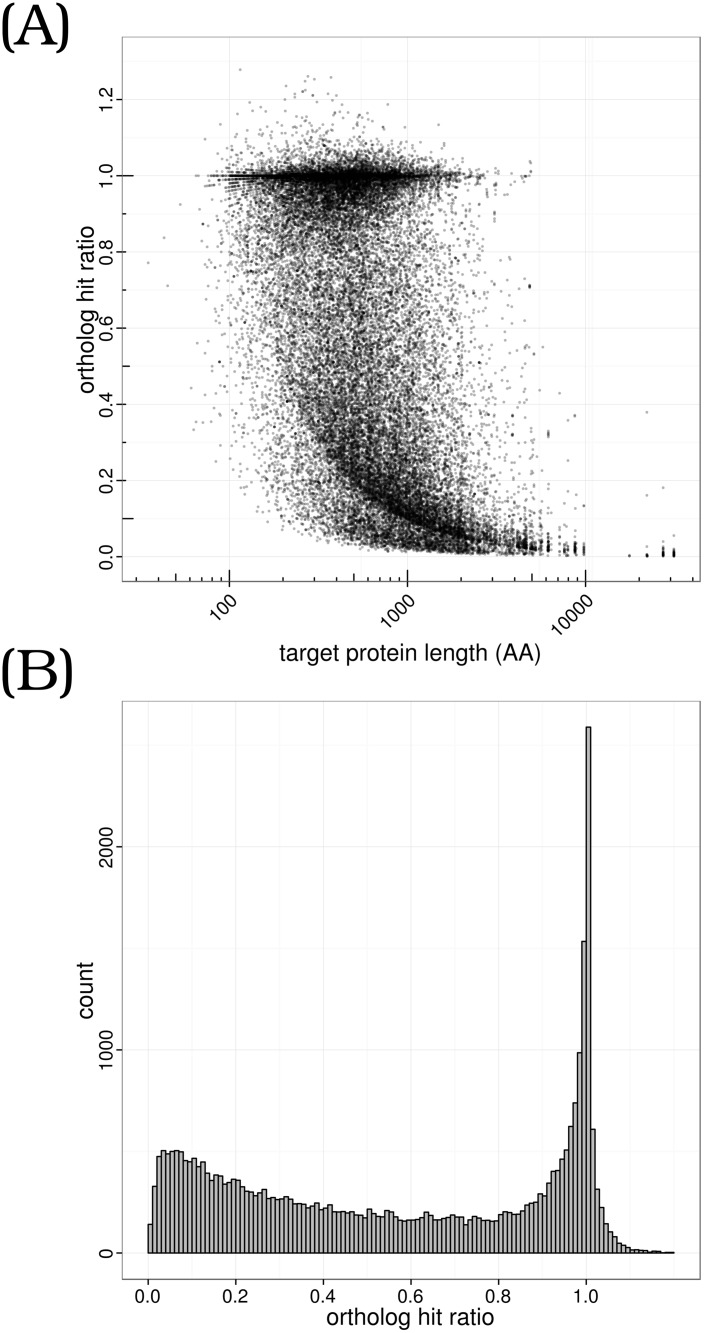
Ortholog hit ratio of transcripts with ORF comparing to zebrafish protein database. (A) ortholog hit ratio against target protein length, showing the relationship of the completeness of transcripts with lengths. (B) Ortholog hit ratio distribution for all transcripts with ORF. A ratio of 1 means the transcript thoroughly covers the target in zebrafish protein database, and a value larger than 1 implies a possible gene extension.

To evaluate the representativeness and completeness of the transcripts with ORF comparing to the whole transcriptome, we used the zebrafish protein database as the benchmark for homologous search. As a result, 33,674 protein sequences from the zebrafish protein database were hit. A related question is to what extent the completeness of transcripts is comparable to their homologous counterparts in zebrafish. To answer this question, the ortholog hit ratio [44] was calculated by dividing the aligned length of a transcript by the total length of the best zebrafish protein hit. By the definition, a transcript with a ortholog hit ratio of 1 implies a full length gene. As shown in Fig 4(A), the ortholog hit ratios of transcripts concentrate around 1.0 and is slightly decreased for long genes, implying good full length assembles for both short and long genes. Of 33,674 transcripts, we found that 10,564 and 17,990 sequences had ratios higher than 0.9 and 0.5, respectively. Notably, we identified 2,830 genes with the ratio larger than 1.0 (Fig 4(B)), suggesting possible elongations in those genes.

### Functional polymorphic SSR detection and analysis

Because of their stability and prevalent distribution along genomes and transcriptomes, SSRs are still widely used in agriculture and population studies [45–47]. SSRs in genes are particularly valuable due to their direct function associations. To develop functional markers, all tandem repeats in the transcriptome were identified using Tandem Repeats Finder (TRF) [32], resulting in the detection of 599,920 tandem repeats in 79,237 transcripts (89.9% of total). Of the total tandem repeats, 494,611 ones were found with two end flanking regions larger than 50 bp for designing PCR primers. CA/TG and CT/AG are the top two most abundant SSR units in the transcriptome of large yellow croaker, followed by two trinucleotide repeats of CTC/GAG and CTG/CAG (S3 Fig). Interestingly, the distribution of dinucleotide repeat units was identical to that in catfish EST analysis [48], however, large yellow croaker and catfish exhibted different trinucleotide unit distributions. This SSR type difference may imply possible marker diversification among species.

**Fig 5:**
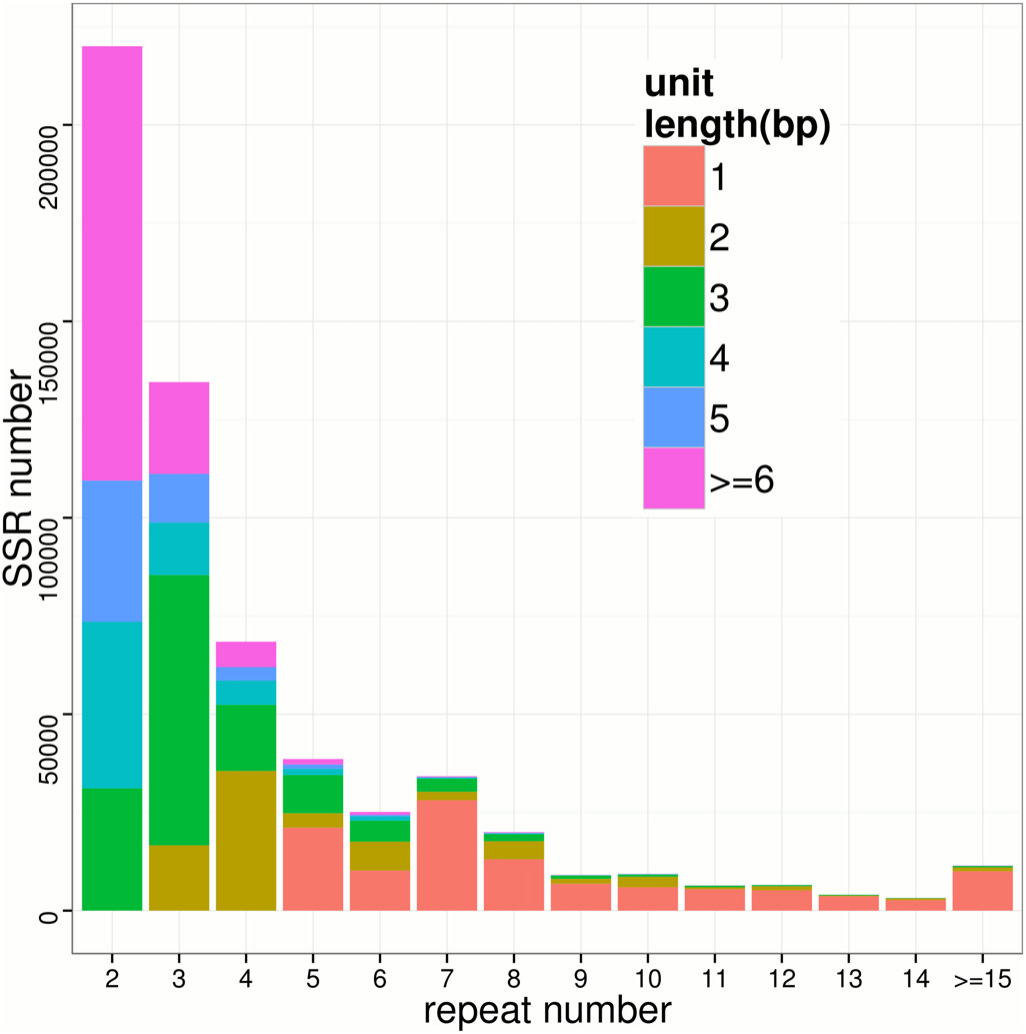
Repeat-numbers distribution of SSRs. The X-axis and Y-axis indicate the repeat number (rounded to the lower whole number) and the number of SSR loci for a given unit length (color key).

To further investigate SSR types in large yellow croaker, we analyzed SSR unit length and repeat number distribution. The overall SSR markers were comprised of 112,349 (19.0%) mononucleotide motifs, 77,056 (13.0%) dinucleotide motifs, 139,707 (23.6%) trinucleotide motifs, 65,392 (11.1%) tetranucleotide motifs, 53,463 (9.0%) pentanucleotide motifs and 65,973 (11.2%) hexanucleotide motifs (S4 Fig). ∼86% (513,940/599,920) SSRs have a unit length no more than 6 bp. The most frequent SSR type in large yellow croaker is trinucleotides (23.6%); more interestingly, 38.4% of overall SSRs are the multiples of trinucleotides and those SSRs are more prominent than their neighbors (S4 Fig). The SSR type distribution is consistent with previous studies that unit repeat number variations of those SSRs are less likely to cause frameshift errors in coding regions [49]. For repeat number, as shown in Fig 5, a huge number of SSRs (78%) have a repeat number lower than 5 and mononucleotide SSRs dominate in high repeat ones (≥5).

**Fig 6:**
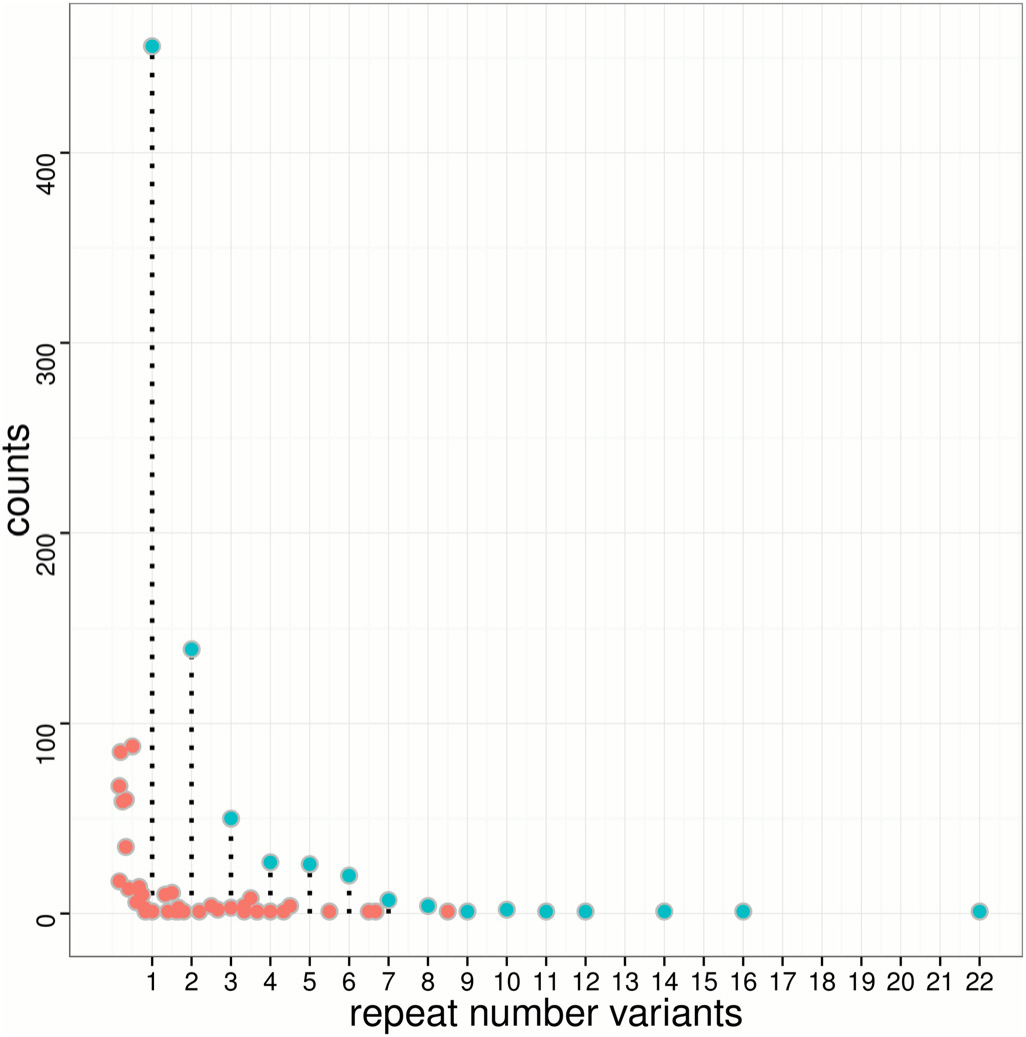
Repeat number variants distribution for detected polymorphic SSRs. The black drops lines for unit variations (green dots) indicates the repeat number variants (X-axis). The non-unit SSR variants (red dots) are in-between of unit ones.

It is essential to detect SSR polymorphism *in silico* and to investigate variants distribution within genes, especially for those transcripts associating with important economic traits [45]. Several methods have been proposed for SSR variant detection from EST sequences; however, it is still challenging to detect SSR polymorphism from short sequence reads [50]. Recently, much effort has been devoted to analyzing repeat variants in genomes of model organisms [49], but studies of non-model organism on transcriptomes have rarely been reported. In this work, lobSTR [33] was employed to call SSR polymorphism in large yellow croaker transcriptome for the first time. With lobSTR pipelines, we identified 1,276 polymorphic SSRs from 872 transcripts. We found that unit lengths for all polymorphic SSRs were less than or equal to 6 bp. Colm *et. al* have reported that around 1 in 20 human proteins are likely to contain SSR polymorphism within coding regions [49]; the frequency is comparable with our case in large yellow croaker. Notably, the majority (57.8%) of variable SSRs are unit mutations, shown as green dots in Fig 6. Repeat number variations by 1 and 2 units are remarkably predominant among all SSR variants, suggesting polymorphism of SSR in transcriptome is likely to be limited to short condense and elongation of proteins.

### Functional SNP/InDel discovery and analysis

Because of their abundance, SNP/InDel offers a useful alternative to SSR in high density marker studies, such as in a fine genetic mapping and quantitative trait locus (QTL) identification [8, 14, 51, 52]. With the rapid cost reduction for NGS, sequence resources are increasing at unprecedented rate for more and more species, enabling much easier and cheaper to develop and to analyze genome- and transcriptome-wide SNP/InDel [11]. Since the sequencing in this work is based on a composite sample of multi individuals, the data provide a valuable resource to investigate the small variant distribution and frequency within the large yellow croaker transcriptome. To this end, we aligned the short sequencing reads to the assembled transcriptome, resulting in alignment for SNP/InDel detection in GATK pipeline [36]. More than 237,000 high-quality SNPs and 24,000 InDels were identified from 30,378 (34%) transcripts, resulting the density of polymorphic sites per 1,000 bp (PS/kb) as 4.28. Of the total detected SNP loci, 666 polymorphic sites multiple alleles because of the composite sample.

In transcripts with ORF, we identified 181,000 (69% of total) SNPs and 17,000 (62% of total) InDels over 18,377 transcripts. The results show that 54% of transcripts with ORF contains SNP/InDel markers, which is significantly higher than that of the total transcriptome (34%). The putative SNPs include 124,079 transitions and 59,636 transversions. The Ts/Tv (transitions / transversions) ratio is estimated as 2.08, which is consistent with previous reports of other aquaculture animals [53]. The detail analysis showed that the transition of G/A and C/T accounted for 33.6% and 33.8%, respectively, and each transversion in G/C, G/T, A/C and A/T accounted for 7.9%∼8.4% of all SNP types. The percentage of base substitutions is also comparable with previous studies [54], implying the reliability of SNP detection and impact analysis in the present work. We note that missense/silent mutation ratio of 0.45 is lower than that reported as ∼1 in Nile tilapia [7]. In protein coding sequences, missense mutations likely cause more deleterious effects to silent ones, therefore the lower ratio implies that transcripts with ORF are under rigorous natural selection. Whether the distinct ratio stems from the species difference or the incomplete genome annotation of large yellow croaker needs further investigations.

**Fig 7:**
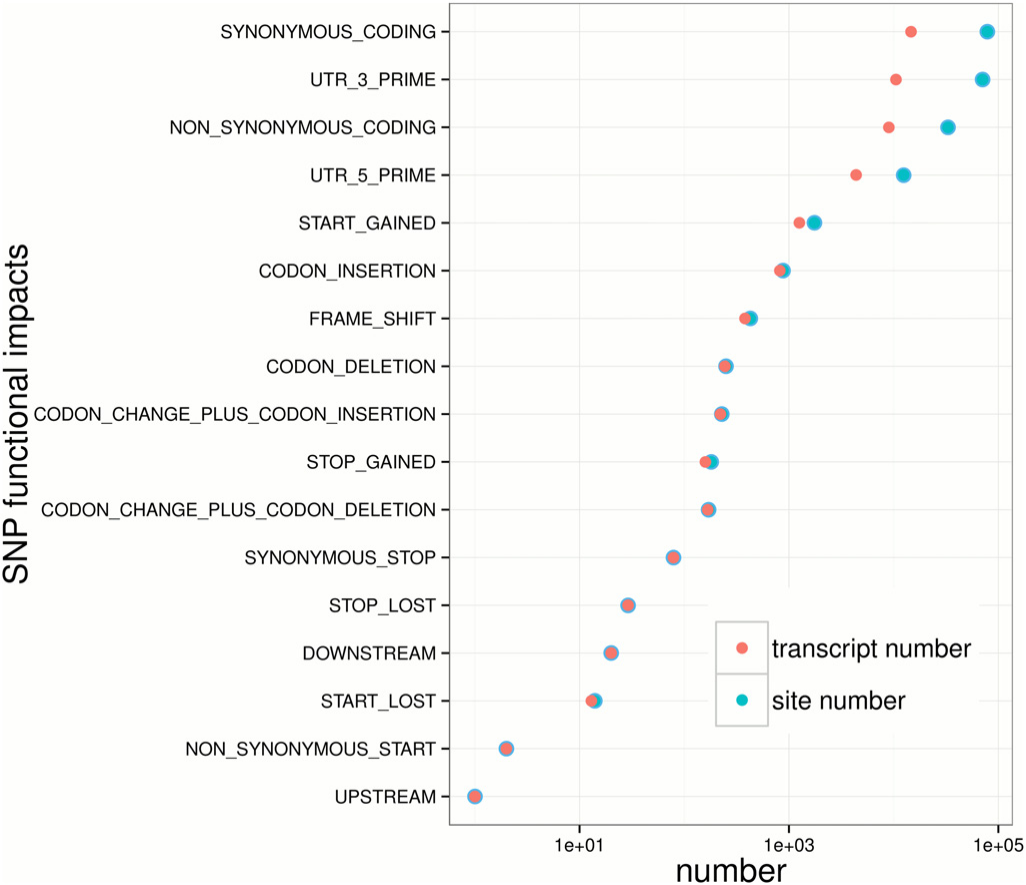
Functional impact analysis for SNPs in transcriptome of large yellow croaker. The Y-axis indicates various functional effect for proteins and the X-axis shows the number of SNP sites (green dots) and SNP-taking transcripts number (red dots) for each functional impact type.

To analyze how SNPs influence gene product, SnpEff 3.6 [55] was employed, as well as the sequence annotation of transcripts, to assess amino acid changes in untranslated region (URT) and ORF. The majority (∼75%) of polymorphic loci are synonymous mutations, followed by changes in 3’ UTRs. SNP and InDel mutations leading to codon insertions, frame-shifts, coding deletions are all below 1,000, implying those mutation types are rather rare comparing to synonymous ones. More interestingly, there are more than 1,000 SNP that potentially lead to a novel initiation codons in the 5’ UTR (start-gained mutations) and the frequency of start-gained SNP is higher than that of stop gained, stop loss and start loss. This suggests that there might be weaker natural selections against start-gained mutations. The recent studies have revealed that many synonymous mutations in genes may also change protein expression levels and structures and thus also be subject to natural selections [56]. However, many scientists believe that more synonymous mutations are nearly neutrally or weakly selected [57]. One piece of evidences is that more synonymous mutations in exon regions are reported than non-synonymous ones, which is also observed in Fig 7. In addition, from Fig 7 we observed that SNP sites and SNP-taking gene numbers are divergent in synonymous, 3” UTR, non-synonymous and 5” UTR mutations, suggesting genes may simultaneously have several SNPs and InDels in those categories.

**Fig 8:**
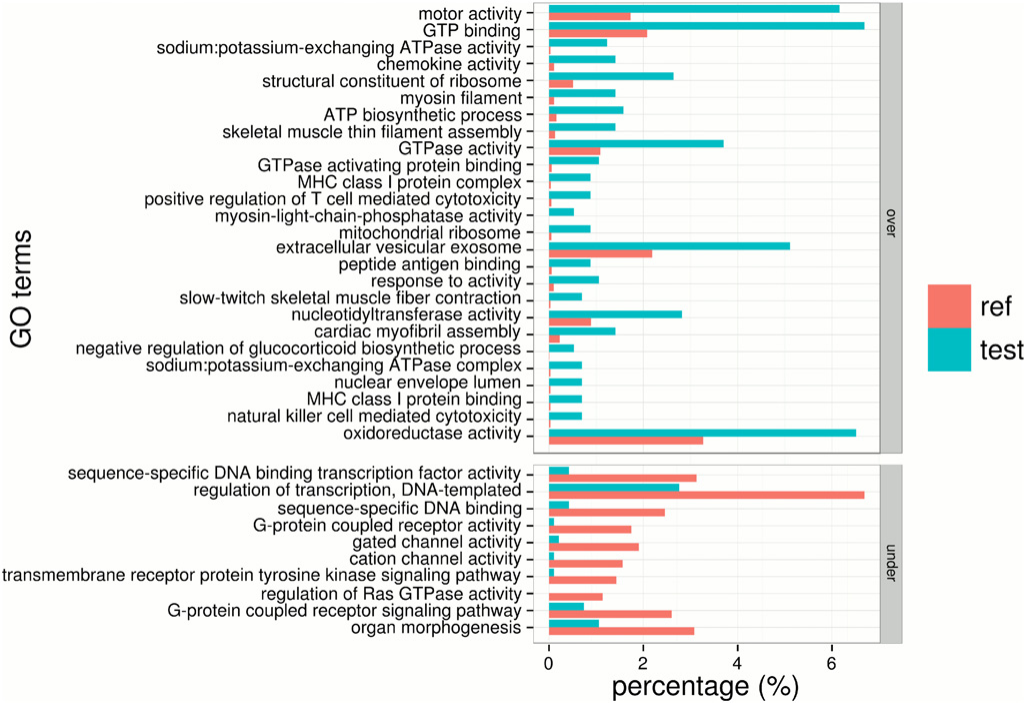
GO enrichment analysis of top 800 highest SNP-rate transcripts. The significance of each GO term is calculated by comparing GO terms of input transcripts (blue) with those in the whole transcriptome (red). The bar length in the figure indicates the percentage of sequences with a specific GO-term. All enriched terms are categorized as over-represented and under-represented groups and sorted by increasing FDR from top to bottom. Note that only GO terms with a FDR ≤ 0.01 are shown.

We analyzed the correlation between gene length and SNP site number and observed a weak correlation coefficient of 0.36 between transcript lengths and SNP sites (S5 Fig), suggesting SNP density within genes were divergent. To investigate functions for SNP-prone transcripts, we performed a GO-enrichment study for the top 800 highest SNP-rate transcripts. We obtained 79 reduced significant GO-terms, with 47 over-represented and 32 under-represented terms (Fig 8). Including the most significant (FDR of 2.2 × 10^−7^) GO-term of motor activity (GO:0003774), we found massively over-represented items associated with the muscular tissue and the cytoskeleton, such as myosin filament (GO:0032982), skeletal muscle thin filament assembly (GO:0030240), slow-twitch skeletal muscle fiber contraction (GO:0031444), structural constituent of cytoskeleton (GO:0005200), cardiac myofilbril assembly (GO:0055003), myofibril (GO:0030016), tublin complex (GO:0045298) and actin filament binding (GO:0051015), actomyosin (GO:0042643) and striate muscle myosin thick filament assembly (GO:0071688). A two-fold explanation is proposed to understand the enrichment result. First, muscular tissues have been reported to contain elastomeric proteins with disordered regions, such as PEVK domains in titin and twitchin [58]. Sequence polymorphism in a disordered region is unlikely to destabilize the whole structure and stability, and thus under weak natural selection. Second, many immunoglobulin domain families in muscle exhibiting similar structures [59] might also show sequence polymorphism. Meanwhile, we also observed several over-represented terms linked with immunology mechanisms, including chemokine activity (GO:0008009), MHC class I protein complex and binding (GO:0042612, GO:0042288), positive regulation of T cell mediated cytotoxicity (GO:0001916), cellular response to interleukin-4 (GO:0071353), peptide antigen binding (GO:0042605) and antigen processing and presentation (GO:0019882). However, GO-terms enriched on sequence-specific DNA bindings, enzyme activities, cell communications and signal pathways were significantly under-represented. Less sequence polymorphisms on those GO-terms may associate with the elaborate regulation and important function of the proteins in those processes of living cells.

## Conclusion

Large yellow croaker is an important economic species in China and Eastern Asian. Based on next-generation sequencing with the Illumina Hiseq2000 platform, we have sequenced mRNA fragments from a composite sample of multiple large yellow croaker individuals from distinct development stages and assembled the transcriptome into 88,103 transcripts. Combining a series of packages and databases, including nt/nr, InterPro, GO and KEGG, 52,782 transcripts have been annotated. 35,321 transcripts without any homologous hits suggest possible non-coding RNA, new genes or species-specific sequences. The gene expression calculation has shown that transcripts exhibit wide expression level distribution. 34,025 transcripts have been identified with potential ORF longer than 300 bp, which have been further annotated with 26 KOG categories.

The polymorphic functional markers have been detected and analyzed on large yellow croaker transcriptome. We have scanned 1,276 polymorphic SSRs, 237,000 SNPs and 24,000 InDels and found a number of polymorphic SSRs and SNPs may be related with essential metabolic pathways and economic traits of large yellow croaker. we have evaluated functional impacts of SNPs on protein sequence and shown that about ∼75% of SNPs lead to synonymous mutations. The functions of high SNP-rate transcripts are enriched in GO-terms related with the muscle tissue, the cytoskeleton and the immunological system, but are under-represented in sequence-specific DNA binding, enzyme activity and cell communications. The detection and functional analysis of polymorphic SSR and SNP in the transcriptome not only sheds light on functional impacts of markers on economic related genes, but also provide the community valuable resources for the further functional marker studies and molecular aided selection of large yellow croaker.

## Supporting Information

S1 Fig The Venn diagram for the homologous search in zebrafish, medaka, pufferfish and stickleback protein database. The cross section number means the shared transcripts in several databases.
(TIFF)

S2 Fig The FPKM measured expression level distribution for all transcripts in the transcriptome. (TIFF)

S3 Fig Top 10 abundant SSR unit type in large yellow croaker. Sequence complementary of SSR have been considered and merged.
(TIFF)

S4 Fig Pie chart for the SSR unit length distribution. The digits outside the pie show nucleotide numbers, namely 1 for mononucleotide, 2 for dinucleotide, 3 for trinucleotide and so on. Note that the percentage for 11 represents all SSRs with the unit length large than 11.
(TIFF)

S5 Fig Pearson correlation analysis of SNP numbers (Y-axis) against transcript lengths (X-axis). The linear fit model (red line) indicates a weak explanation (r = 0.36) of the SNP diversification from transcript lengths.
(TIFF)

## References

[1] Wang J, Ai Q, Mai K, Xu H, Zuo R. Dietary chromium polynicotinate enhanced growth performance, feed utilization, and resistance to Cryptocaryon irritans in juvenile large yellow croaker (*Larmichthys crocea*). Aquaculture. 2014;432:321–326. 10.1016/j.aquaculture.2014.05.027

[2] Liu X, Zhao G, Cai M, Wang Z. Estimated genetic parameters for growth-related traits in large yellow croaker Larimichthys crocea using microsatellites to assign parentage. J Fish Biol. 2013;82(1):34–41. 10.1111/j.1095-8649.2012.03472.x 23331136

[3] Wang X, Li Y, Hou C, Gao Y, Wang Y. Physiological and molecular changes in large yellow croaker (*Pseudosciaena crocea R.*) with high-fat diet-induced fatty liver disease. Aquac Res. 2015;46(2):272–282. 10.1111/are.12176

[4] Liu J, Yu Z, Lin Y, Chen H, Xie W. Studies on the Pseudomonas disease of large yellow croaker. Marine Sci. 2004;2:001.

[5] Mu Y, Ding F, Cui P, Ao J, Hu S, Chen X. Transcriptome and expression profiling analysis revealed changes of multiple signaling pathways involved in immunity in the large yellow croaker during Aeromonas hydrophila infection. BMC genomics. 2010;11(1):506 10.1186/1471-2164-11-506 20858287 PMC2997002

[6] Garber M, Grabherr M, Guttman M, Trapnell C. Computational methods for transcriptome annotation and quantification using RNA-seq. Nat Methods. 2011;8(6):469–477. 10.1038/nmeth.1613 21623353

[7] Xia J, Wan Z, Ng Z, Wang L, Fu G, Lin G, et al Genome-wide discovery and in silico mapping of gene-associated SNPs in Nile tilapia. Aquaculture. 2014;432:67–73. 10.1016/j.aquaculture.2014.04.028

[8] Cui J, Wang H, Liu S, Zhu L, Qiu X, Jiang Z, et al SNP Discovery from Transcriptome of the Swimbladder of Takifugu rubripes. PloS ONE. 2014;9(3):e92502 10.1371/journal.pone.0092502 24651578 PMC3961390

[9] Zhang L, Li L, Zhu Y, Zhang G, Guo X. Transcriptome analysis reveals a rich gene set related to innate immunity in the eastern oyster (*Crassostrea virginica*). Mar Biotechnol. 2014;16(1):17–33. 10.1007/s10126-013-9526-z 23907648

[10] Zhou Z, Dong Y, Sun H, Yang A, Chen Z, Gao S, et al Transcriptome sequencing of sea cucumber (*Apostichopus japonicus*) and the identification of gene-associated markers. Mol Ecol Resour. 2014;14(1):127–138. 10.1111/1755-0998.12147 23855518

[11] Xu J, Ji P, Zhao Z, Zhang Y, Feng J, Wang J, et al Genome-wide SNP discovery from transcriptome of four common carp strains. PloS ONE. 2012;7(10):e48140 10.1371/journal.pone.0048140 23110192 PMC3482183

[12] Sun L, Liu S, Wang R, Jiang Y, Zhang Y, Zhang J, et al Identification and analysis of genome-wide SNPs provide insight into signatures of selection and domestication in channel catfish (*Ictalurus punctatus*). PloS ONE. 2014;9(10):e109666 10.1371/journal.pone.0109666 25313648 PMC4196944

[13] Liu S, Zhou Z, Lu J, Sun F, Wang S, Liu H, et al Generation of genome-scale gene-associated SNPs in catfish for the construction of a high-density SNP array. BMC genomics. 2011;12(1):53 10.1186/1471-2164-12-53 21255432 PMC3033819

[14] Kucuktas H, Wang S, Li P, He C, Xu P, Sha Z, et al Construction of genetic linkage maps and comparative genome analysis of catfish using gene-associated markers. Genetics. 2009;181(4): 1649–1660. 10.1534/genetics.108.098855 19171943 PMC2666527

[15] Liu F, Sun F, Xia JH, Li J, Fu GH, Lin G, et al A genome scan revealed significant associations of growth traits with a major QTL and GHR2 in tilapia. Sci Rep. 2014;4:7256 10.1038/srep07256 25435025 PMC4248272

[16] Wang X, Li L, Zhu Y, Du Y, Song X, Chen Y, et al Oyster shell proteins originate from multiple organs and their probable transport pathway to the shell formation front. PloS ONE. 2013;8(6):e66522 10.1371/journal.pone.0066522 23840499 PMC3686672

[17] Zhou P, Zhang Z, Wang Y, Zou Z, Xie F. EST analysis and identification of gonad-related genes from the normalized cDNA library of large yellow croaker, *Larimichthys crocea* . Comp Biochem Physiol Part D Genomics Proteomics. 2010;5(2):89–97. 10.1016/j.cbd.2010.01.002 20403775

[18] Mu Y, Li M, Ding F, Ding Y, Ao J, Hu S, et al De novo characterization of the spleen transcriptome of the large yellow croaker (*Pseudosciaena crocea*) and analysis of the immune relevant genes and pathways involved in the antiviral response. PloS ONE. 2014;9(5):e97471 10.1371/journal.pone.0097471 24820969 PMC4018400

[19] Quilang J, Wang S, Li P, Abernathy J, Peatman E, Wang Y, et al Generation and analysis of ESTs from the eastern oyster, Crassostrea virginica Gmelin and identification of microsatellite and SNP markers. BMC genomics. 2007;8(1):157 10.1186/1471-2164-8-157 17559679 PMC1919373

[20] Ye H, Wang X, Gao T, Wang Z. EST-derived microsatellites in Pseudosciaena crocea and their applicability to related species. Acta Oceanologica Sinica. 2010;29(6):83–91. 10.1007/s13131-010-0079-y

[21] Lü Z, Li H, Liu L, Cui W, Hu X, Wang C. Rapid development of microsatellite markers from the large yellow croaker (*Pseudosciaena crocea*) using next generation DNA sequencing technology. Biochem Syst Ecol. 2013;51:314–319. 10.1016/j.bse.2013.09.019

[22] Bioinformatics B. FASTQC: A quality control tool for high throughput sequence data. Cambridge, UK: Babraham Institute; 2012.

[23] Grabherr M, Haas B, Yassour M, Levin J, Thompson D, Amit I, et al Full-length transcriptome assembly from RNA-Seq data without a reference genome. Nat Biotechnol. 2011;29(7):644–652. 10.1038/nbt.1883 21572440 PMC3571712

[24] Li W, Godzik A. Cd-hit: a fast program for clustering and comparing large sets of protein or nucleotide sequences. Bioinformatics. 2006;22(13):1658–1659. 10.1093/bioinformatics/btl158 16731699

[25] Altschul S, Madden T, Schäffer A, Zhang J, Zhang Z, Miller W, et al Gapped BLAST and PSI-BLAST: a new generation of protein database search programs. Nucleic Acids Res. 1997;25(17):3389–3402. 10.1093/nar/25.17.3389 9254694 PMC146917

[26] Conesa A, Gotz S, García-gómez J, Terol J, Talón M, Robles M. Blast2GO: a universal tool for annotation, visualization and analysis in functional genomics research. Bioinformatics. 2005;21(18):3674–3676. 10.1093/bioinformatics/bti610 16081474

[27] Li B, Dewey C. RSEM: accurate transcript quantification from RNA-Seq data with or without a reference genome. BMC bioinformatics. 2011;12(1):323 10.1186/1471-2105-12-323 21816040 PMC3163565

[28] Langmead B, Trapnell C, Pop M, Salzberg S, et al Ultrafast and memory-efficient alignment of short DNA sequences to the human genome. Genome Biol. 2009;10(3):R25 10.1186/gb-2009-10-3-r25 19261174 PMC2690996

[29] Haas B, Papanicolaou A, Yassour M, Grabherr M, Blood P, Bowden J, et al *De novo* transcript sequence reconstruction from RNA-seq using the Trinity platform for reference generation and analysis. Nat Protoc. 2013;8(8):1494–1512. 10.1038/nprot.2013.084 23845962 PMC3875132

[30] Bateman A, Coin L, Durbin R, Finn R, Hollich V, Griffiths-jones S, et al The Pfam protein families database. Nucleic Acids Res. 2004;32(suppl 1):D138–D141. 10.1093/nar/gkh121 14681378 PMC308855

[31] Wu S, Zhu Z, Fu L, Niu B, Li W. WebMGA: a customizable web server for fast metagenomic sequence analysis. BMC genomics. 2011;12(1):444 10.1186/1471-2164-12-444 21899761 PMC3180703

[32] Benson G. Tandem repeats finder: a program to analyze DNA sequences. Nucleic Acids Res. 1999;27(2):573 10.1093/nar/27.2.573 9862982 PMC148217

[33] Gymrek M, Golan D, Rosset S, Erlich Y. lobSTR: a short tandem repeat profiler for personal genomes. Genome Res. 2012;22(6):1154–1162. 10.1101/gr.135780.111 22522390 PMC3371701

[34] Li H, Durbin R. Fast and accurate short read alignment with Burrows-Wheeler transform. Bioinformatics. 2009;25(14):1754–1760. 10.1093/bioinformatics/btp324 19451168 PMC2705234

[35] Li H, Handsaker B, Wysoker A, Fennell T, Ruan J, Homer N, et al The sequence alignment/map format and SAMtools. Bioinformatics. 2009;25(16):2078–2079. 10.1093/bioinformatics/btp352 19505943 PMC2723002

[36] Mckenna A, Hanna M, Banks E, Sivachenko A, Cibulskis K, Kernytsky A, et al The Genome Analysis Toolkit: a MapReduce framework for analyzing next-generation DAN sequencing data. Genome Res. 2010;20(9):1297–1303. 10.1101/gr.107524.110 20644199 PMC2928508

[37] Fisher R. A Preliminary Linkage Test with Agouti and Undulated Mice. Heredity. 1949;3:229–241. 10.1038/hdy.1949.16 18149092

[38] Benjamini Y, Hochberg Y. Controlling the false discovery rate: a practical and powerful approach to multiple testing. J R Stat Soc Series B Stat Methodol. 1995;p. 289–300.

[39] Ye J, Fang L, Zheng H, Zhang Y, Chen J, Zhang Z, et al WEGO: a web tool for plotting GO annotations. Nucleic Acids Res. 2006;34(suppl 2):W293–W297. 10.1093/nar/gkl031 16845012 PMC1538768

[40] Trapnell C, Williams B, Pertea G, Mortazavi A, Kwan G, Van baren M, et al Transcript assembly and quantification by RNA-Seq reveals unannotated transcripts and isoform switching during cell differentiation. Nat Biotechnol. 2010;28(5):511–515. 10.1038/nbt.1621 20436464 PMC3146043

[41] Arava Y, Wang Y, Storey J, Liu C, Brown P, Herschlag D. Genome-wide analysis of mRNA translation profiles in Saccharomyces cerevisiae. Proc Natl Acad Sci USA. 2003;100(7):3889–3894. 10.1073/pnas.0635171100 12660367 PMC153018

[42] Chen Y, Capeyrou R, Humbert O, Mouffok S, Al kadri Y, Lebaron S, et al The telomerase inhibitor Gno1p/PINX1 activates the helicase Prp43p during ribosome biogenesis. Nucleic Acids Res. 2014;42(11):7330–7345. 10.1093/nar/gku357 24823796 PMC4066782

[43] Gish W, States D. Identification of protein coding regions by database similarity search. Nat Genet. 1993;3(3):266–272. 10.1038/ng0393-266 8485583

[44] T o’neil S, Dzurisin J, Carmichael R, Lobo N, Emrich S, Hellmann J. Population-level transcriptome sequencing of nonmodel organisms Erynnis propertius and Papilio zelicaon. BMC genomics. 2010;11(1):310 10.1186/1471-2164-11-310 20478048 PMC2887415

[45] Duran C, Singhania R, Raman H, Batley J, Edwards D. Predicting polymorphic EST-SSRs in silico. Mol Ecol Resour. 2013;13(3):538–545. 10.1111/1755-0998.12078 23398650

[46] Song W, Li Y, Zhao Y, Liu Y, Niu Y, Pang R, et al Construction of a high-density microsatellite genetic linkage map and mapping of sexual and growth-related traits in half-smooth tongue sole (*Cynoglossus semilaevis*). PloS ONE. 2012;7(12):e52097 10.1371/journal.pone.0052097 23284884 PMC3527371

[47] Wang W, Hui JH, Chan TF, Chu KH. De novo transcriptome sequencing of the snail *Echinolittorina malaccana*: identification of genes responsive to thermal stress and development of genetic markers for population studies. Mar Biotechnol. 2014;16(5):547–559. 10.1007/s10126-014-9573-0 24825364

[48] Serapion J, Kucuktas H, Feng J, Liu Z. Bioinformatic mining of type I microsatellites from expressed sequence tags of channel catfish (*Ictalurus punctatus*). Mar Biotechnol. 2004;6(4):364–377. 10.1007/s10126-003-0039-z 15136916

[49] T o’dushlaine C, Edwards R, Park S, Shields D. Tandem repeat copy-number variation in protein-coding regions of human genes. Genome Biol. 2005;6(8):R69 10.1186/gb-2005-6-8-r69 16086851 PMC1273636

[50] Cao M, Balasubramanian S, Bodén M. Sequencing technologies and tools for short tandem repeat variation detection. Brief Bioinform. 2014;p. bbu001. 10.1093/bib/bbu001 24504770

[51] Wang S, Sha Z, Sonstegard TS, Liu H, Xu P, Somridhivej B, et al Quality assessment parameters for EST-derived SNPs from catfish. BMC genomics. 2008;9(1):450 10.1186/1471-2164-9-450 18826589 PMC2570692

[52] Houston R, Taggart J, Cézard T, Bekaert M, Lowe N, Downing A, et al Development and validation of a high density SNP genotyping array for Atlantic salmon (*Salmo salar*). BMC genomics. 2014;15(1):90 10.1186/1471-2164-15-90 24524230 PMC3923896

[53] Yu Y, Wei J, Zhang X, Liu J, Liu C, Li F, et al SNP Discovery in the Transcriptome of White Pacific Shrimp Litopenaeus vannamei by Next Generation Sequencing. PloS ONE. 2014;9(1):e87218 10.1371/journal.pone.0087218 24498047 PMC3907553

[54] Wondji C, Hemingway J, Ranson H. Identification and analysis of single nucleotide polymorphisms (SNPs) in the mosquito Anopheles funestus, malaria vector. BMC genomics. 2007;8(1):5 10.1186/1471-2164-8-5 17204152 PMC1781065

[55] Reumers J, Schymkowitz J, Ferkinghoff-borg J, Stricher F, Serrano L, Rousseau F. SNPeffect: a database mapping molecular phenotypic effects of human non-synonymous coding SNPs. Nucleic Acids Res. 2005;33(suppl 1):D527–D532. 10.1093/nar/gki086 15608254 PMC540040

[56] Sauna Z, Kimchi-sarfaty C. Understanding the contribution of synonymous mutations to human disease. Nat Rev Genet. 2011;12(10):683–691. 10.1038/nrg3051 21878961

[57] Zeng K, Charlesworth B. Studying patterns of recent evolution at synonymous sites and intronic sites in Drosophila melanogaster. J Mol Evol. 2010;70(1):116–128. 10.1007/s00239-009-9314-6 20041239

[58] Linke W, Kulke M, Li H, Fujita-becker S, Neagoe C, Manstein D, et al PEVK domain of titin: an entropic spring with actin-binding properties. J Struct Biol. 2002;137(1):194–205. 10.1006/jsbi.2002.4468 12064946

[59] Tskhovrebova L, Trinick J. Titin: properties and family relationships. Nat Rev Mol Cell Biol. 2003;4(9):679–689. 10.1038/nrm1198 14506471

