# Supplementary figures and images for: Functional Marker Detection and Analysis on a Comprehensive Transcriptome of Large Yellow Croaker by Next Generation Sequencing
Source: PLoS One. 2015 Apr 24;10(4):e0124432. doi: 10.1371/journal.pone.0124432 (PMC4409302; doi:10.1371/journal.pone.0124432)

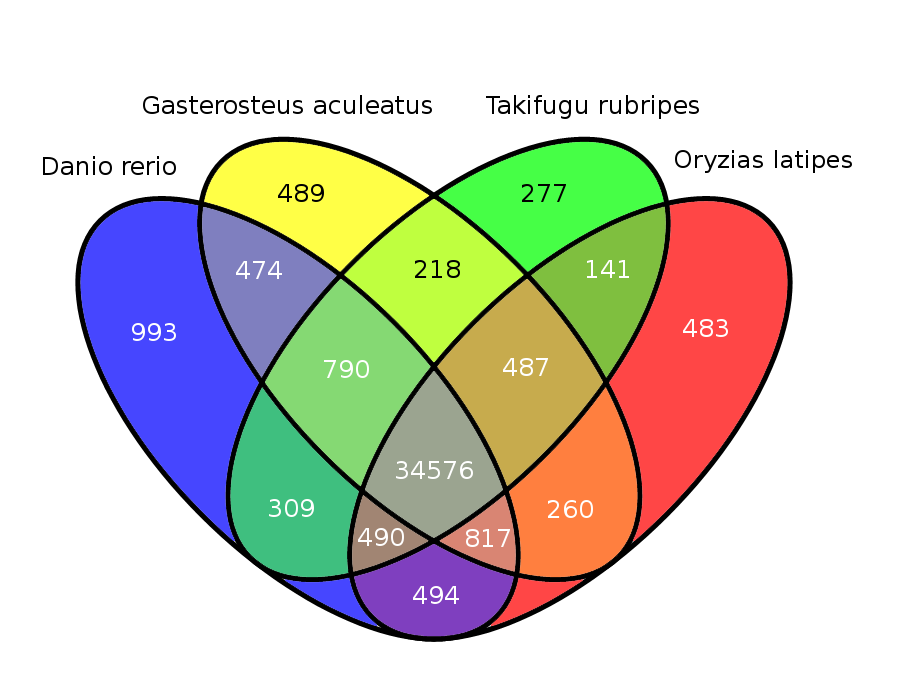

Supplement: S1 Fig — The cross section number means the shared transcripts in several databases. (TIFF) [file pone.0124432.s001.tiff]

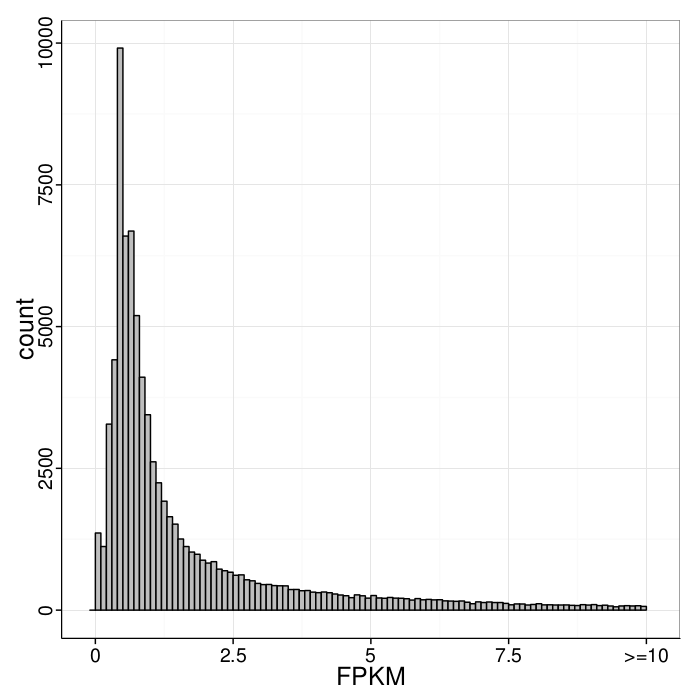

Supplement: S2 Fig — (TIFF) [file pone.0124432.s002.tiff]

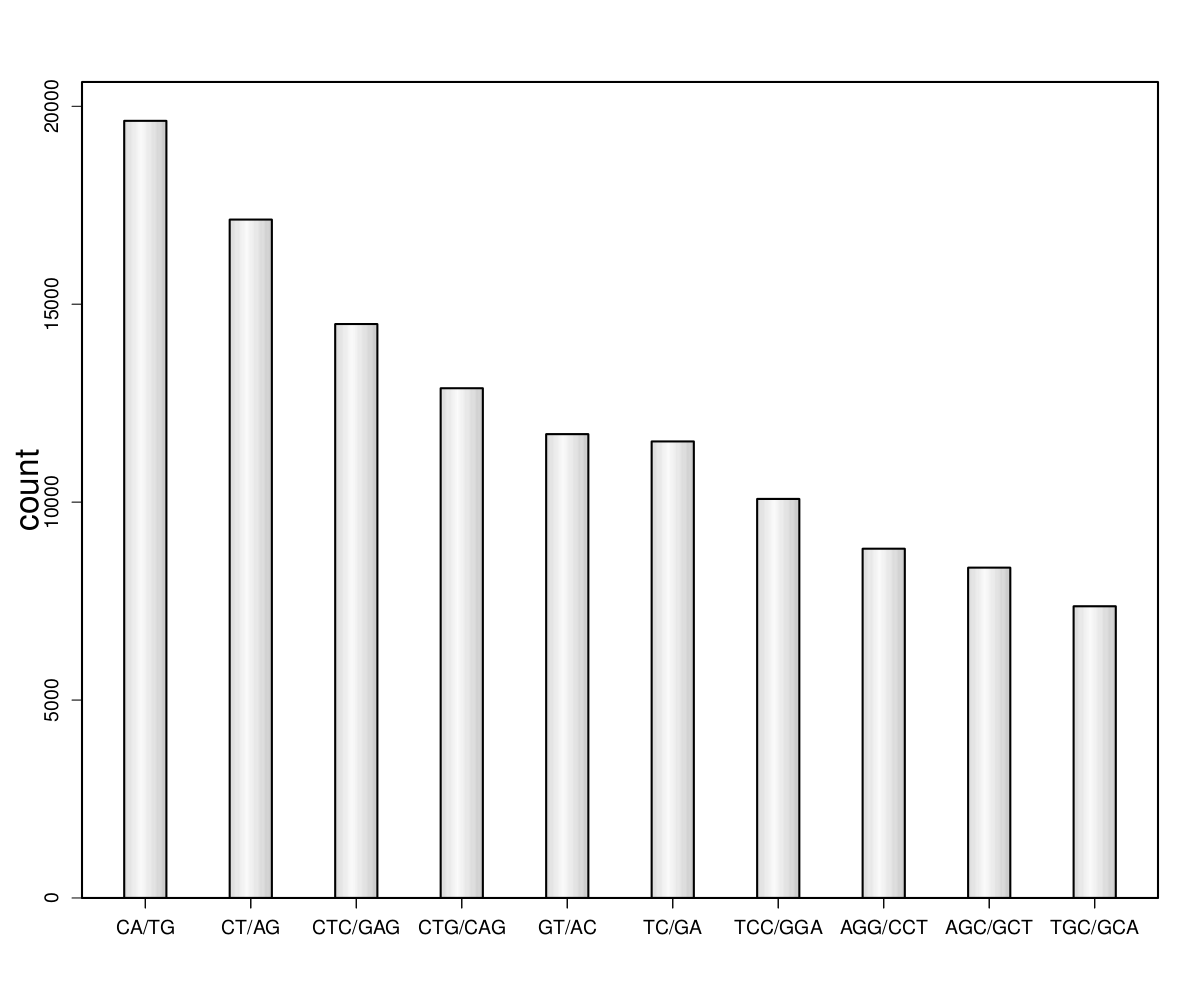

Supplement: S3 Fig — Sequence complementary of SSR have been considered and merged. (TIFF) [file pone.0124432.s003.tiff]

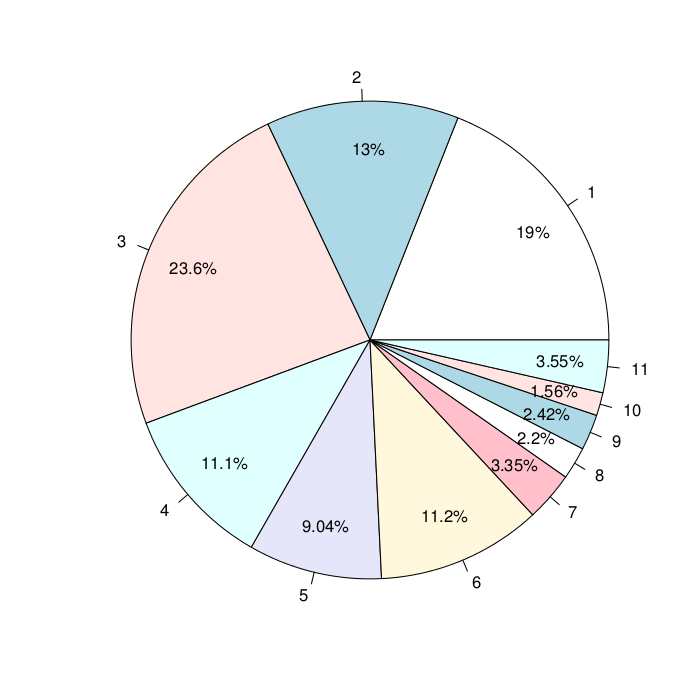

Supplement: S4 Fig — The digits outside the pie show nucleotide numbers, namely 1 for mononucleotide, 2 for dinucleotide, 3 for trinucleotide and so on. Note that the percentage for 11 represents all SSRs with the unit length large than 11. (TIFF) [file pone.0124432.s004.tiff]

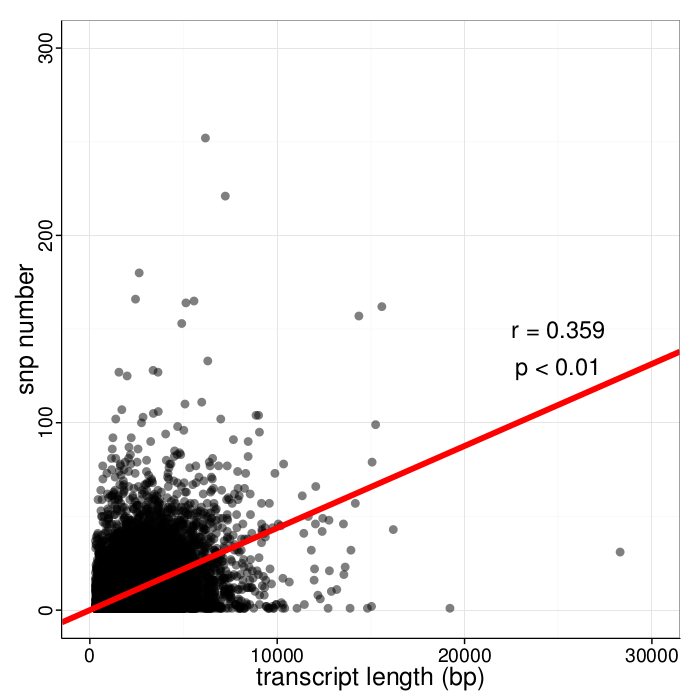

Supplement: S5 Fig — The linear fit model (red line) indicates a weak explanation (r = 0.36) of the SNP diversification from transcript lengths. (TIFF) [file pone.0124432.s005.tiff]
